# Anisotropic Hardening of TRIP780 Steel Sheet: Experiments and Analytical Modeling

**DOI:** 10.3390/ma16041414

**Published:** 2023-02-08

**Authors:** Jizhen Wang, Miao Han, Chong Zhang, Hasib Md Abu Rayhan, Xvyang Li, Yanshan Lou

**Affiliations:** 1 School of Mechanical Engineering, Xi’an Jiao Tong University, 28 Xianning West Road, Xi’an 710049, China; 2 Aviation Key Laboratory of Science and Technology on Structure Impact Dynamics, Aircraft Strength Research Institute of China, Xi’an 710065, China

**Keywords:** anisotropic hardening, yield function, convexity

## Abstract

By combining experimental and theoretical models, this research investigates the anisotropic hardening behaviors of TRIP780 steel. The specimens of TRIP780 steel were subjected to uniaxial tensile and bulging tests under different loading conditions to obtain hardening data. The experimental results show that the strength and plastic deformation of TRIP780 steel vary with the loading directions, which indicates that TRIP780 steel has anisotropy characteristics. In this paper, the dichotomous method is used to ensure the convexity of the Chen-coupled quadratic and non-quadratic (CQN) function. Comparing the predictions of the hardening behavior of the TRIP780 steel sheet by the Yld2000-2d, Stoughton-Yoon’2009 and Chen-CQN functions, the results show that the Chen-CQN function exhibits the advantages of simple numerical implementation and a more realistic prediction of yield stress compared to the former two, respectively. Comparing the prediction of Chen-CQN function with the experimental hardening data, the results show that the deviation between the experimental data and the experimental response given by the function is always within 3%, and this function maintains an accurate prediction under different stress states, indicating that the Chen-CQN yield function has accuracy and flexibility for the characterization of the yield surface of TRIP780 steel.

## 1. Introduction

With excellent mechanical properties, steel is widely used in the automotive industry. Advanced High-Strength Steel (AHSS) gradually came into our view due to the increasing concern for energy and environmental issues. The application of AHSS in the automotive industry can meet strength requirements while making possible lighter vehicles, thus providing an important way to save energy and reduce emissions [1]. Transformation-induced plasticity (TRIP) steel is a type of AHSS. During the forming of TRIP steels, the residual austenite, under the influence of applied stress or strain, is transformed into the hard martensitic phase, which hardens the steel and increases its ductility, thereby increasing its plasticity and strength at the same time [2]. At present, TRIP780 steel has had an active role in lightweight design in the automotive industry. The selection of a suitable yield function according to the hardening behavior of TRIP780 steel helps to assure the reliability of the numerical simulation of steel forming and thus meets the safety requirements of practical applications.

To study the complex yield behavior of metals, many yield criteria have been published. The Hill48 yield criterion [3] is one of the pioneering results and is based on the Huber-von Mises yield function with the addition of four anisotropic parameters, and provides an accurate prediction of uniaxial tensile and equibiaxial tensile hardening curves along rolling direction (RD), transverse direction (TD) and normal direction (ND). Subsequently, to improve the accuracy of the yield equation to characterize the hardening behavior, the linear transformation of the stress tensor was used to increase the number of anisotropic parameters, and more anisotropic yield criteria emerged. Barlat et al. [4], respectively, put forward yield criteria to describe anisotropic metal sheets such as aluminum alloy sheets. Barlat et al. [5] developed the anisotropic yield function based on linear transformation to more accurately characterize the anisotropic behavior of metals and alloys under the spatial stress state. Lou [6] developed a symmetric yield function considering the strength difference (SD) effect under the associated flow rule, which accurately predicted the anisotropic and asymmetric hardening behavior of the metals. Aretz and Barlat [7] developed two yield functions for orthotropic metals under plane and three-dimensional (3D) stress states. Lou [8] effectively improved the Yld2004-18p function [6] by a reduced linear transformation tensor and successfully illustrating the yielding and anisotropic deformation of metals with moderate anisotropy. The anisotropic function developed by Hu et al. [9] described the SD effect along RD, DD and TD.

When the above yield functions are applied to numerical simulation in practice, they are mainly used to represent the hardening behavior under plane stress due to the high complexity of the model under spatial loading. The anisotropic yield functions in a form of stress invariants are relatively simple for plasticity modelling under spatial loading, so they are widely applied in numerical simulations [10]. Drucker [11] modified the Huber-von Mises yield criterion by adding the third stress invariant. Cazacu and Barlat [12] extended the application of the yield function by replacing the stress invariant of the Drucker yield function with the corresponding orthogonal anisotropic form. Yoon et al. [13] proposed an asymmetric yield function with the first invariant, which accurately characterized the SD effect of the materials in the process of tension and compression. Yoshida et al. [14] proposed a 3D yield function that can guarantee the convexity of the yield surface, which can well describe the anisotropic hardening behavior of sheet metals such as steel plates. Lou and Yoon [15] achieved an effective differentiation of the anisotropic hardening behavior of metals with a body-centered cubic (BCC) and face-centered cubic (FCC) structure by correcting for the effect of the third invariant. Recently, Hu et al. [16] analytically calibrated the parameters of the ploy6 yield criterion [17] and applied it to AA5182-O to evaluate the characterization of the anisotropic behaviors. Hu and Yoon [18] developed a new constitutive model to characterize anisotropic plastic flow under tension and compression accurately without interpolation methods.

The anisotropy parameters in the yield criterion need to be calibrated analytically to predict anisotropic behavior since there are differences in hardening between different loading directions and conditions under proportional loadings [19]. Stoughton and Yoon [19] responded by substituting the data on the hardening behavior of the uniaxial and equibiaxial tension of RD, DD, and TD for the values found in the Hill48 yield function, and the resulting simulated scenario matched the hardening behavior in reality. Because the accuracy of the Stoughton and Yoon function as a quadratic function in describing shear and plane strains is not satisfactory, Lee et al. [20] proposed the CQN model, which has a yield function that is the result of coupling the above-mentioned quadratic and Hershey–Hosford yield functions. Park et al. [21] explained the asymmetric yield surface evolution behavior over a large strain and temperature range by introducing scaling and asymmetric functions into Stoughton and Yoon. Hou et al. [22] adapted the quadratic yield function of the CQN model and presented anisotropic pressure-sensitive constitutive equations by adding a weighted pressure term. Hu et al. [23] constructed an anisotropic hardening model with analytical parameters by coupling the fourth-order polynomial (ploy4) yield function with the Hosford yield function. Hou et al. [24] described the hardening characteristics of anisotropic materials directly using hardening curves under uniaxial tension and compression and the equibiaxial tension condition along RD, DD and TD. 

Many results on hardening and yielding have also been presented recently. Wu et al. [25] characterized the yield behavior of the Mg-Gd-Y alloy under the coupling effect of a temperature and stress state. Hou et al. [26] developed an asymmetric anisotropic yield criterion portraying the capture of the anisotropic characteristics of different metallic materials in the manufacturing of automotive sheet metals. The yield criterion developed by Hu et al. takes into account the pure shear stresses along 0°, 45°and 90° from RD simultaneously, enabling prediction for both pure shear and tension stress states [27]. Du et al. [28] compared the predictions of anisotropic behaviors for the asymmetric yield criterion under associated flow rule (AFR) and non-associated flow rule (NAFR). Hou et al. developed a yield criterion under NAFR to characterize the anisotropic evolution of sheet metal under plane strain (PS) conditions [29]. The anisotropic hardening function proposed by Chen et al. [30] can accurately account for the differences in the yielding behavior of metals with a BCC and FCC structure. The computational speed of the numerical simulation of Chen’ function has been improved compared to the CQN model, while the computational accuracy of both is very close. Lou et al. [31] proposed a stress-invariant yield function that can be used to accurately simulate the strain-hardening behavior of metal with a BCC, FCC and hexagonal close packed (HCP) structure under different stress states, and the convexity of the function can be analyzed using a simple numerical analysis method.

In this paper, the hardening behavior of TRIP780 steel was studied by the tensile test and bulging test with loading directions of RD, DD, and TD. Additionally, three anisotropic yield functions were chosen to describe the anisotropic hardening behavior of TRIP780 steel, and their convexity was guaranteed by the dichotomy method. The effectiveness of the three yield functions in predicting the hardening curves of TRIP780 steel was compared; since the Chen-CQN model has a simple form for numerical application and can give a highly precise prediction, the function was used to predict the plastic deformation of TRIP780 steel. The performance of the Chen-CQN function was further illustrated by comparing the hardening curves predicted by the function with the experimental results. 

## 2. Experiments

**Figure 1:**
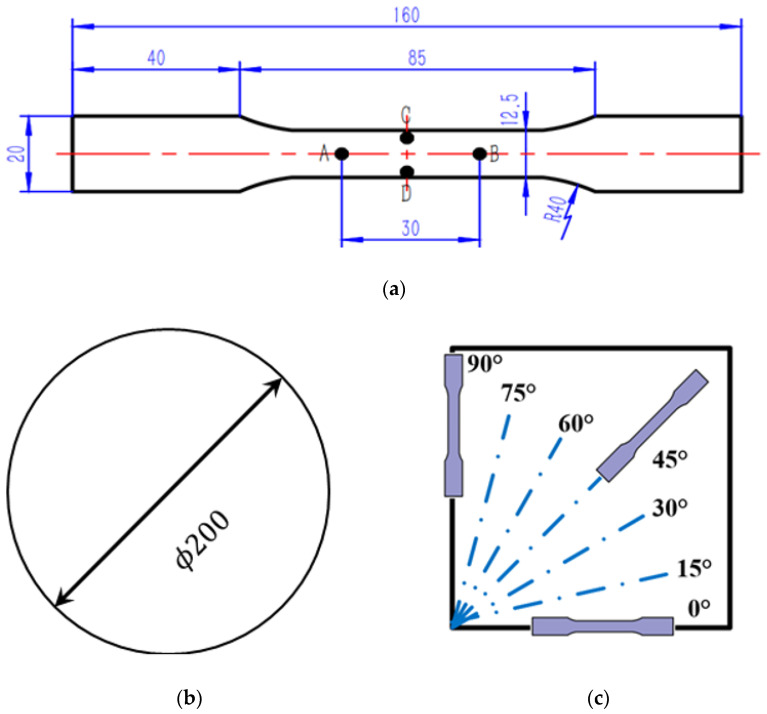
Experiment design for anisotropic hardening of TRIP780 [unit: mm]. (**a**) dogbone specimen; (**b**) bulging specimen; (**c**) different loading directions.

This section aims to collect hardening date under uniaxial and equibiaxial (EB) tension to assess the performance of the different yield criterions for TRIP780 steel. The TRIP780 steel sheet used in the experiment is produced by Baoshan Iron & Steel Co., Ltd. (Shanghai, China), and its chemical composition is shown in Table 1. The dogbone specimen in Figure 1a and the bulging specimen in Figure 1b were employed to conduct the tensile test and the bulging test equivalent to the equibiaxial tension, respectively. Thickness of the specimens was 3 mm. As shown in Figure 1c, the dogbone was stretched at every 15° from RD.

The uniaxial tensile test was performed using GB/T 228.1-2021 [32] as the standard. Uniaxial tensile tests were performed at room temperature and quasi-static conditions. Experimental setup consisted of the XTOP digital image correlation (DIC) system and the ETM104B electronic universal testing machine. DIC system was responsible for collecting real-time images of the specimen during the experiment so as to measure and analyze the deformation of the dogbone.

The crosshead speed should be set according to the parallel length of the dogbone specimen and the target strain rate before conducting the experiment. The strain rate under quasi-static conditions was 0.001/s, so crosshead speed was set to 3.6 mm/min for this experiment. Since the deformation at both ends of the parallel region in the middle of the specimen is not guaranteed to be uniform, the region with uniform deformation is selected to set the extensometer to obtain the correct measurement results. The length of this area is generally equal to 1/2 to 2/3 of the parallel length. In Figure 1, points A and B marked on the specimen are the endpoints of the extensometer in the axial direction, and points C and D are the endpoints of the extensometer in the width direction. Axial extensometer gauge length is 30 mm, and the stroke was calculated by axial gauge length change measured during experiment. At least 3 tests should be conducted along each loading directions to ensure the reliability of the test results.

**Figure 2:**
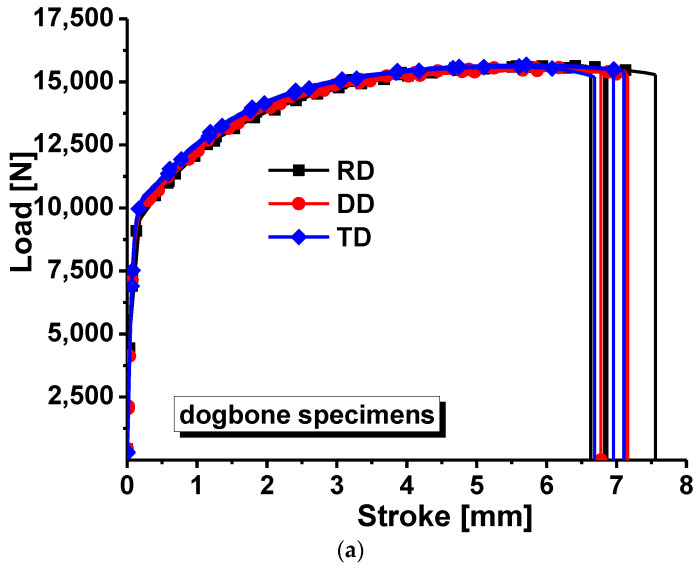

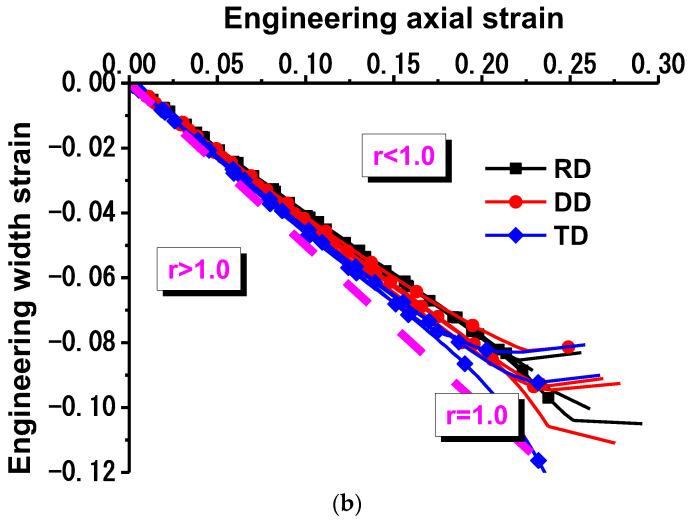
Experimental results of the dogbone specimens along different directions: (**a**) load–stroke curves; (**b**) axial strain–width strain for the calculation of R-values.

After the uniaxial tensile tests were completed, the most representative tensile test data along RD, DD and TD were selected to make the load–stroke curve as shown in Figure 2a. Figure 2b gives the axial strain and width strain relationship curves of the dogbone specimens along three loading directions and the calculated results of the incremental ratio in the direction of width and thickness (r-value). Comparing the experimental results between different groups, it can be found that the experiments are easily reproducible. The maximum forces along RD, DD and TD in Figure 2a increase accordingly, and r-values of different loading directions in Figure 2b are different, respectively, indicating the anisotropy of the strength and plastic deformation of TRIP780 steel. The load–stroke curves of each group were selected for the best repeatability, and the true stress–strain curves were calculated for the uniaxial tensile test along three loading directions: 0°, 45°, and 90° away from RD.

**Figure 3:**
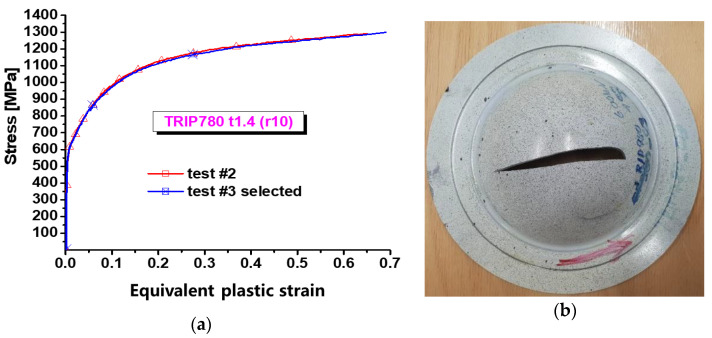
Experimental results of equibiaxial tension from bulging tests (blank holding force 600 kN): (**a**) stress–strain curves of equibiaxial tension; (**b**) deformed specimen.

The hardening data under equibiaxial tension were obtained by bulging test. Stress–strain curves are shown in Figure 3a, which show that the experiments have good repeatability. Figure 3b reveals that the specimens underwent inhomogeneous deformation, indicating the anisotropic plastic deformation of the TRIP780 steel. The Swift–Voce hardening function was used to describe the hardening curves; the expression of this hardening function is as follows:

(1) $$\sigma =\alpha K{\left(\varepsilon_{0}+\varepsilon_{p}\right)}^{n}+(1-\alpha )\left(A-(A-B)\exp\left(-C\varepsilon_{p}\right)\right)$$

where $\varepsilon_{0}$ is yield strain, m is work hardening coefficient, *A* is saturation stress, *B* is yield stress, and *K* and *C* are material constants.

**Figure 4:**
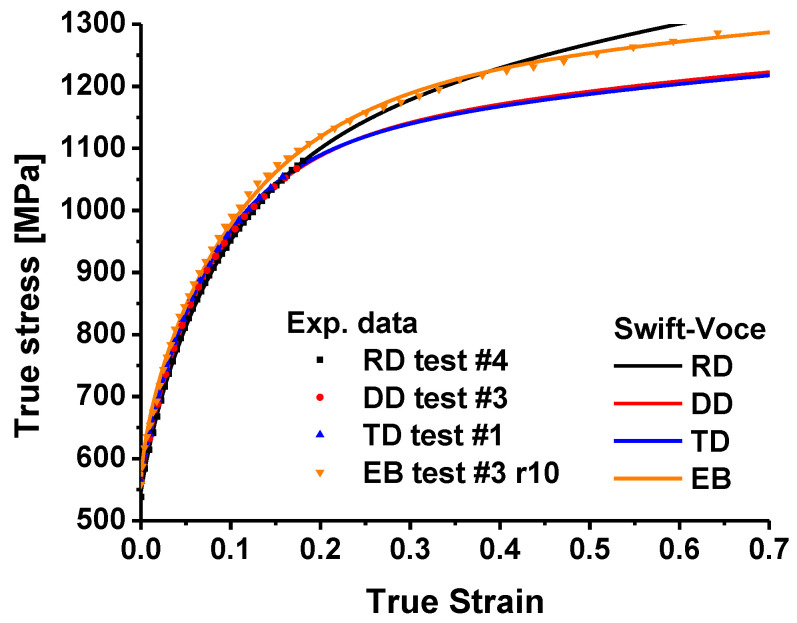
Comparison of the stress–strain curves between experiments and Swift–Voce hardening law at uniaxial and equibiaxial tension.

The coefficients of the Swift–Voce function are shown in Table 2. As shown in Figure 4, the calibrated hardening curves are compared with the stress–strain curves of the above two tensile experiments. It is easy to see that the calibrated curves have a good fitting effect on the test data, showing that the Swift–Voce hardening function has high accuracy for characterizing the hardening behavior of TRIP780 steel, and the difference of the true stress along RD, DD and TD indicates that TRIP780 steel is anisotropic.

**Figure 5:**
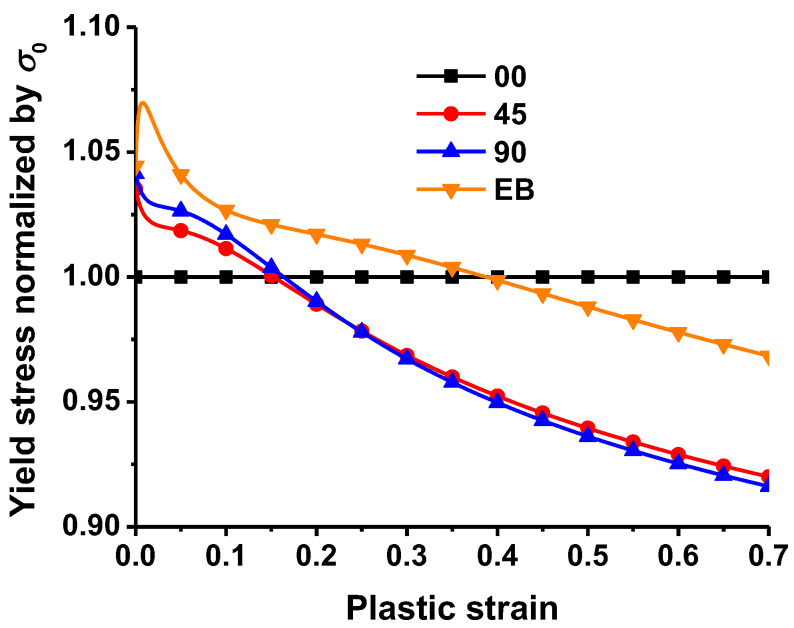
Anisotropic hardening behavior of the alloy.

The hardening curves of the two experiments were normalized by that along RD, as shown in Figure 5. Through the indication that the loading direction has a significant impact on the steel’s hardening behavior and by the further demonstration that TRIP780 steel has a strong anisotropic hardening characteristic, it can be seen that the hardening curves of the steel show significant differences with the change of the loading direction.

## 3. Anisotropic Hardening Functions

To determine suitable yield functions for the numerical simulation of TRIP780, Yld2000-2d [4], Stoughton-Yoon’2009 [19], and the newly proposed coupled quadratic-nonquadratic [24] yield functions are selected for comparison.

### 3.1. Yld2000-2d Function

The expression of the Yld2000-2d function for the plane strain problem is as follows:

(2) $$\bar{\phi }{\left(\sigma \right)}_{Yld2000}={\left(\frac{\emptyset^{\prime }+\emptyset^{\prime \prime }}{2}\right)}^{\frac{1}{m}}$$

(3) $$err=\overset{l}{\underset{i=1}{\sum }}w_{i}{\left(\frac{\sigma_{i}^{exp}}{\sigma_{i}^{pred}}-1\right)}^{2}+\overset{n}{\underset{j=1}{\sum }}w_{j}{\left(\frac{r_{j}^{exp}}{r_{j}^{pred}}-1\right)}^{2}$$

where $\emptyset^{\prime }$ and $\emptyset^{\prime \prime }$ are two isotropic functions. Both *i* and *j* represent experimental data points used to optimize material parameters—typically, tensile yield stresses and *r*-values of uniaxial tension along 0°, 45°, and 90° and equibiaxial tension. $X_{1,2}^{\prime \prime }$ are the principal values of the two linearly transformed deviatoric stress tensors $X_{}^{\prime }$ and $X_{}^{\prime \prime }$, respectively. The exponent m value is recommended to be six for metal with a BCC structure, and eight for metal with an FCC structure. The two tensors $X_{}^{\prime }$ and $X_{}^{\prime \prime }$ are calculated as follows:

(4) $$X^{\prime }=C^{\prime }s=C^{\prime }T\sigma =L^{\prime }\sigma ,\;\emptyset^{\prime \prime }={\left|2X_{2}^{\prime \prime }+X_{1}^{\prime \prime }\right|}^{m}+{\left|2X_{1}^{\prime \prime }+X_{2}^{\prime \prime }\right|}^{m}$$

(5) $$C_{}^{\prime }=\left[\begin{array}{ccc} C_{11}^{\prime } & C_{12}^{\prime } & 0\\ C_{21}^{\prime } & C_{22}^{\prime } & 0\\ 0 & 0 & C_{66}^{\prime } \end{array}\right],C_{}^{\prime \prime }=\left[\begin{array}{ccc} C_{11}^{\prime \prime } & C_{12}^{\prime \prime } & 0\\ C_{21}^{\prime \prime } & C_{22}^{\prime \prime } & 0\\ 0 & 0 & C_{66}^{\prime \prime } \end{array}\right],T=\left[\begin{array}{ccc} 2/3 & -1/3 & 0\\ -1/3 & 2/3 & 0\\ 0 & 0 & 1 \end{array}\right]$$

where s is the deviatoric stress tensor, $C_{}^{\prime }$ and $C_{}^{\prime \prime }$ are two different fictitious matrices and ***T*** is the transformation matrix. The coefficients of $L^{\prime }$ and $L^{\prime \prime }$ are expressed as follows:

(6) $$\left[\begin{array}{c} \begin{array}{c} L_{11}^{\prime }\\ L_{12}^{\prime }\\ L_{21}^{\prime } \end{array}\\ L_{22}^{\prime }\\ L_{66}^{\prime } \end{array}\right]=\left[\begin{array}{ccc} 2/3 & 0 & 0\\ -1/3 & 0 & 0\\ 0 & -1/3 & 0\\ 0 & 2/3 & 0\\ 0 & 0 & 1 \end{array}\right]\left[\begin{array}{c} \begin{array}{c} \alpha_{1}\\ \alpha_{2} \end{array}\\ \alpha_{7} \end{array}\right],\;\left[\begin{array}{c} \begin{array}{c} L_{11}^{\prime \prime }\\ L_{12}^{\prime \prime }\\ L_{21}^{\prime \prime } \end{array}\\ L_{22}^{\prime \prime }\\ L_{66}^{\prime \prime } \end{array}\right]=\frac{1}{9}\left[\begin{array}{ccccc} -2 & 2 & 8 & -2 & 0\\ 1 & -4 & -4 & 4 & 0\\ 4 & -4 & -4 & 1 & 0\\ -2 & 8 & 2 & -2 & 0\\ 0 & 0 & 1 & 0 & 9 \end{array}\right]\left[\begin{array}{c} \begin{array}{c} \alpha_{3}\\ \alpha_{4} \end{array}\\ \alpha_{5}\\ \alpha_{6}\\ \alpha_{8} \end{array}\right]$$

where $\alpha_{1}$~$\alpha_{8}$ are eight anisotropic coefficients that are usually calibrated by stress values $\sigma_{0/45/90}$, $\sigma_{b}$ and r-values $\sigma_{0/45/90}$, $r_{b}$ under equal-biaxial tensions. The minimization function used for calibration is as below:

(7) $$err=\overset{l}{\underset{i=1}{\sum }}w_{i}{\left(\frac{\sigma_{i}^{exp}}{\sigma_{i}^{pred}}-1\right)}^{2}+\overset{n}{\underset{j=1}{\sum }}w_{j}{\left(\frac{r_{j}^{exp}}{r_{j}^{pred}}-1\right)}^{2}$$

where $\sigma_{i}^{exp}$, $r_{j}^{exp}$ and $\sigma_{i}^{pred}$, $r_{j}^{pred}$ are experimental and predicted stress and r-values, respectively. Variables $w_{i}$ and $w_{j}$ are weighting factors for the stress and r-values.

### 3.2. Stoughton-Yoon’2009 Function

In the case of proportional loading of the sheet, the hardening curves along different loading directions will differ. This result suggests that the shape of the yield surface is only approximately constant during yield hardening for materials with anisotropic characteristics, contradicting the assumption of constant yield surface shape for each isotropic yielding model. To address this issue, Stoughton-Yoon’2009 replaced the original anisotropy parameter of the Hill48 yield criterion with the hardening curves for 0°, 45°, 90° and the isotropic biaxial tension conditions in the rolling direction in the plane stress state.

(8) $$f_{s}\left(\sigma ,\bar{\lambda }\right)=\left(\frac{\sigma_{11}}{\sigma_{0}^{2}\left(\bar{\lambda }\right)}-\frac{\sigma_{22}}{\sigma_{90}^{2}\left(\bar{\lambda }\right)}\right)\left(\sigma_{11}-\sigma_{22}\right)+\frac{\sigma_{11}\sigma_{22}-\sigma_{12}\sigma_{21}}{\sigma_{b}^{2}\left(\bar{\lambda }\right)}+\frac{4\sigma_{12}\sigma_{21}}{\sigma_{45}^{2}\left(\bar{\lambda }\right)}$$

where $\bar{\lambda }$ is the effective plastic strain and $\sigma_{0}\left(\bar{\lambda }\right)$, $\sigma_{45}\left(\bar{\lambda }\right)$, $\sigma_{90}\left(\bar{\lambda }\right)$ and $\sigma_{b}\left(\bar{\lambda }\right)$ correspond to the hardening data under uniaxial tension conditions along RD, DD and TD and equibiaxial tension conditions.

### 3.3. Newly Proposed Coupled Quadratic-Nonquadratic Function

The Stoughton-Yoon’2009 yield criterion has good accuracy in portraying anisotropic hardening under uncorrelated flow rules, and the CQN model has good performance in describing the curvature of the yield surface of metals under plane tension conditions. Chen et al. introduced the *c* parameter to the Cazacu’2018 function for materials with BCC and FCC structures to characterize anisotropic hardening of the materials with BCC and FCC structures under proportional loading conditions.

The Cazacu’2018 yielding function using stress invariants $J_{2}^{}$ and $J_{3}^{}$ takes the following form:

(9) $${\bar{\sigma }}_{f}\left(\sigma_{ij}\right)=a{\left(J_{2}^{4}-cJ_{2}J_{3}^{2}\right)}^{1/8}$$

where parameter *a* can be expressed as

(10) $$a={\left(\frac{81\times 27}{27-4c}\right)}^{\frac{1}{8}}$$

The value of *c* has an important influence on the evolution of the yield surface, and values of 1.5776 and 2.5116 for parameter *c* in the Cazacu’2018 yield function calibrate the yielding behavior of materials with a BCC and FCC structure, respectively.

Based on the CQN model, the Cazacu’2018 yield function can be extended to anisotropic hardening by replacing the $J_{2}$ of the Cazacu’2018 yield function with the transformed Hill48 yield function in the S-Y 2009 function.

(11) $${\bar{\sigma }}_{f}\left(\sigma_{ij}\right)=b{\left[J_{2}\left(J_{2}^{3}-cJ_{3}^{2}\right)\right]}^{1/8}$$

(12) $$f_{c}\left(\sigma ,\bar{\lambda }\right)={\left[f_{Hill48}\left(\sigma ,\bar{\lambda }\right)\cdot f_{\mathrm{Drucker}}\left(\sigma \right)\right]}^{\frac{1}{8}}=1$$

with

(13) $$\begin{array}{rr} & f_{Hill48}\left(\sigma ,\bar{\lambda }\right)=F\left(\bar{\lambda }\right){\left(\sigma_{22}-\sigma_{33}\right)}^{2}+G\left(\bar{\lambda }\right){\left(\sigma_{33}-\sigma_{11}\right)}^{2}+H\left(\bar{\lambda }\right){\left(\sigma_{11}-\sigma_{22}\right)}^{2}\\ & +2L\left(\bar{\lambda }\right)\sigma_{23}^{2}+2M\left(\bar{\lambda }\right)\sigma_{31}^{2}+2N\left(\bar{\lambda }\right)\sigma_{12}^{2}\\ & f_{\mathrm{Drucker}}\left(\sigma \right)=a\left(J_{2}^{3}-cJ_{3}^{2}\right) \end{array}$$

where Equation (13) is a rearrangement of the Cazacu (2018) yield function, $f_{Hill48}\left(\sigma ,\bar{\lambda }\right)$ is an anisotropic hardening function based on Hill48, and $f_{\mathrm{Drucker}}\left(\sigma \right)$ is the Drucker function.

It is not difficult to find that the value of a can be determined, so the calculation of the original Hill48 yield function for the anisotropic hardening parameters can be extended to the calculation of the parameters of f Hill48. Function $f_{Hill48}$ is calculated as follows:

(14) $$\begin{array}{l} F\left(\bar{\lambda }\right)=\frac{1}{2}\left(\frac{1}{{\left[\sigma_{90}\left(\bar{\lambda }\right)\right]}^{8}}+\frac{1}{{\left[\sigma_{ND}\left(\bar{\lambda }\right)\right]}^{8}}-\frac{1}{{\left[\sigma_{0}\left(\bar{\lambda }\right)\right]}^{8}}\right)\\ G\left(\bar{\lambda }\right)=\frac{1}{2}\left(\frac{1}{{\left[\sigma_{0}\left(\bar{\lambda }\right)\right]}^{8}}+\frac{1}{{\left[\sigma_{ND}\left(\bar{\lambda }\right)\right]}^{8}}-\frac{1}{{\left[\sigma_{90}\left(\bar{\lambda }\right)\right]}^{8}}\right)\\ H\left(\bar{\lambda }\right)=\frac{1}{2}\left(\frac{1}{{\left[\sigma_{0}\left(\bar{\lambda }\right)\right]}^{8}}+\frac{1}{{\left[\sigma_{90}\left(\bar{\lambda }\right)\right]}^{8}}-\frac{1}{{\left[\sigma_{ND}\left(\bar{\lambda }\right)\right]}^{8}}\right)\\ L\left(\bar{\lambda }\right)=\frac{1}{2{\left[\tau_{yz}\left(\bar{\lambda }\right)\right]}^{8}}\\ M\left(\bar{\lambda }\right)=\frac{1}{2{\left[\tau_{xz}\left(\bar{\lambda }\right)\right]}^{8}}\\ N\left(\bar{\lambda }\right)=\frac{1}{2{\left[\tau_{xy}\left(\bar{\lambda }\right)\right]}^{8}} \end{array}$$

where $\sigma_{0}\left(\bar{\lambda }\right)$, $\sigma_{90}\left(\bar{\lambda }\right)$ and $\sigma_{ND}\left(\bar{\lambda }\right)$ are the hardening curves for uniaxial tension conditions along the RD, TD, and normal direction (ND), and $\tau_{yz}\left(\bar{\lambda }\right)$, $\tau_{xz}\left(\bar{\lambda }\right)$ and $\tau_{xy}\left(\bar{\lambda }\right)$ are the shear hardening curves in the *yz*, *xz* and *xy* planes.

## 4. Convexity Analysis

According to the previous analysis in Figure 5, it is not hard to find that TRIP780 steel shows obvious anisotropic characteristics of uniaxial tension under different loading conditions, and there are some differences in the hardening behavior of TRIP780 steel under the two stress states of uniaxial and equibiaxial tension. Furthermore, the difference in plastic strain between stress states reduces the possibility of the material exhibiting surface convexity. Therefore, the convexity of the yield function needs to be further investigated in this paper.

**Figure 6:**
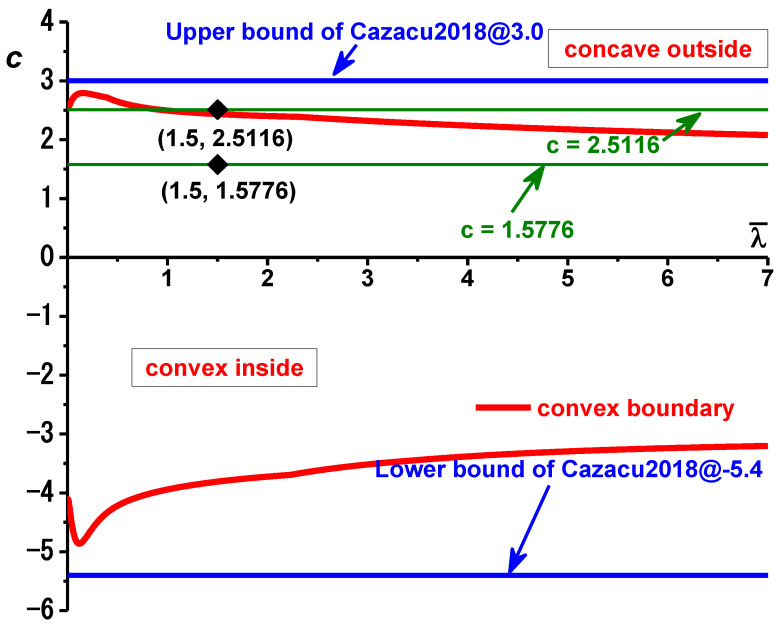
Convex domain computed by GINCA of the Chen-CQN model for TRIP780.

The parameter c controls the curvature of the yield loci in the Chen-CQN yield model, and the equivalent plastic strain $\bar{\lambda }$ plays a significant role in the anisotropic material parameters in it, so the convexity of the function’s yield surface is determined by c and $\bar{\lambda }$. In this regard, the dichotomous method is used in this paper to analyze the convexity of the function. The convex domain, i.e., the area between the two red solid lines, is delineated according to the parameter c of the function and the different strains $\bar{\lambda }$ in the case of plane stress and equibiaxial loading conditions, as shown in Figure 6. It is obvious that the critical value of the parameter c, which allows the yield function to satisfy the requirement of external convexity, varies with the value of $\bar{\lambda }$. This is because the value of the plastic strain will affect the anisotropy parameter in the function, causing the shape of the yield surface to change continuously. Considering that the anisotropic Chen-CQN model is transformed into the Cazacu’2018 model when the material is isotropic, the convex domain divided by the two is compared, and the solid blue line in the figure is the boundary of the convex domain of the Cazacu’2018 model. It is not difficult to find that the convex domain of the Chen-CQN function is contained by the convex domain of the Cazacu’2018 hardening function, and the reason for this phenomenon is that the anisotropy of the material increases the possibility of distortion of the yield surface, which reduces the convex domain of the function.

**Figure 7:**
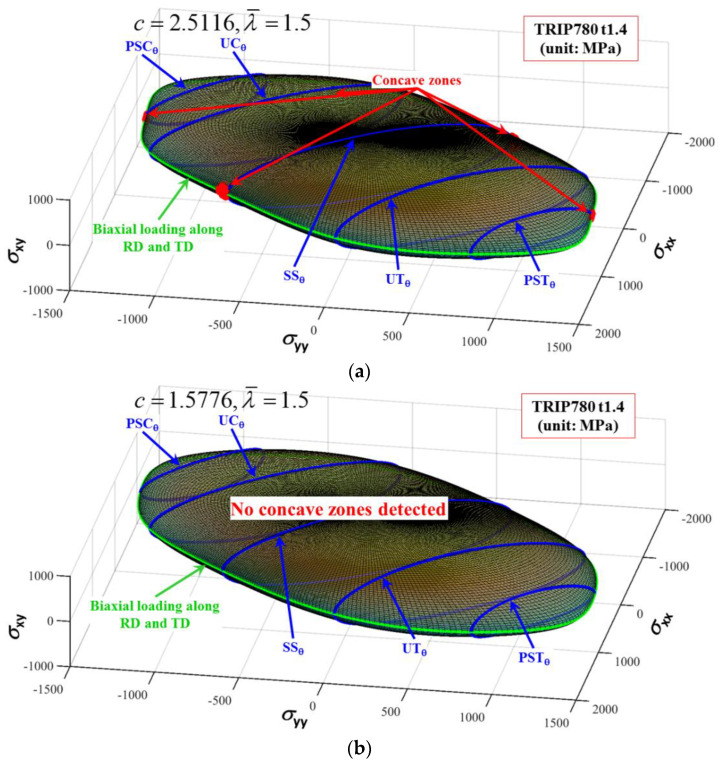
Convexity analysis of the Chen-CQN surface at two special points in Figure 7: (**a**) concave; and (**b**) convex.

TRIP780 steel is an alloy with a BCC structure, so as long as $\bar{\lambda }$ is within the range of [0, 7] in the figure, the hardening law is always in the convex domain, which can ensure that the yield surface is convex. Figure 7 shows the images of the yield surface corresponding to two points with coordinate values of $\bar{\lambda }$ = 1.5, c = 2.5116 and $\bar{\lambda }$ = 1.5, c = 1.5776, where the depressed areas are marked with red balls. Points $\bar{\lambda }$ = 1.5 and c = 2.5116 are outside the convex domain, and the corresponding yield surface is concave. As can be seen in Figure 7a, half of the yield surface of this point contains five concave domains, all of which are distributed around the plane strain state. The point $\bar{\lambda }$ = 1.5, c = 1.5776 is located inside the convex domain, and the yield surface corresponding to this point is found to be convex without any concave domains, as seen in Figure 7b.

## 5. Results

In order to investigate more deeply whether the yield function can accurately characterize the anisotropic behavior of TRIP780 steel, the predicted yield trajectories of TRIP780 by the Yld2000-2d, S-Y 2009 and Chen-CQN functions were compared with the experimental results. Table 3 lists the parameter values of the Yld2000-2d model corresponding to the different plastic strains of the material, which were calibrated by the hardening curve data of TRIP780. Table 3 shows that the coefficients of the Yld2000-2d function at different strain levels are different, which leads to the complex form of numerical application.

**Figure 8:**
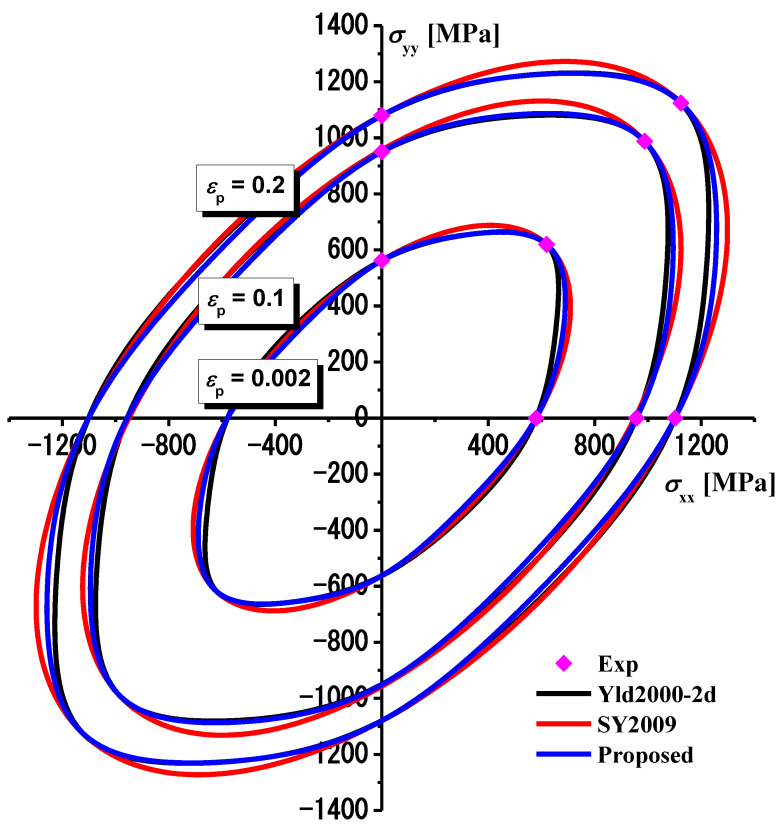
Comparison of yield surface evolution between experiments and prediction under biaxial loading along RD and DD (*c* = 2.0).

The evolution of the yield surface of TRIP780 steel at different equivalent plastic strains predicted by the three yield functions shown in Figure 8 was compared with the corresponding results of the experiments. The yield stresses predicted by the three functions have almost no deviation from the experimental hardening data along RD, DD and TD. However, the predictions of yield stress of the Yld2000-2d and Chen-CQN yield functions are smaller than that of the S-Y 2009 yield function. The result of this comparison indicates that the predictions of the Yld2000-2d and Chen-CQN functions are more consistent with the actual situation. As the Yld2000-2d function has different parameters at different strains, the Chen-CQN yield function ensures an accurate characterization of the yield surface while having a simple numerical application form. Therefore, the Chen-CQN yield function has higher flexibility when characterizing the plastic deformation of TRIP780 steel.

**Figure 9:**
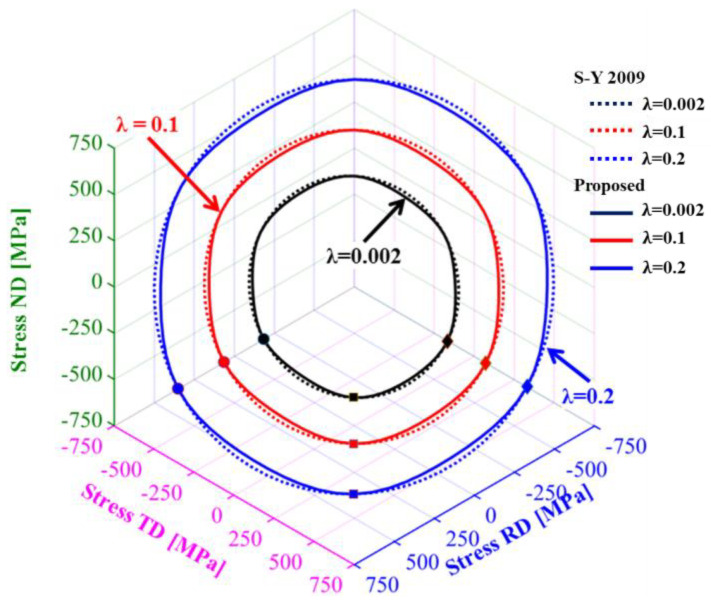
Comparison of yield surface evolution between experiments and prediction on π-plane.

Figure 9 depicts the prediction of the yield surface of TRIP780 under spatial loading on the π-plane by the S-Y 2009 and Chen-CQN models. The points in the figure represent experimental data along RD, DD and TD obtained through uniaxial tensile experiments. It is not difficult to find that the Chen-CQN function is able to predict lower yield stresses at different stress states compared to the Yld2000-2d function, so the Chen-CQN function has a higher accuracy when predicting the yield surface of TRIP780 steel.

**Figure 10:**
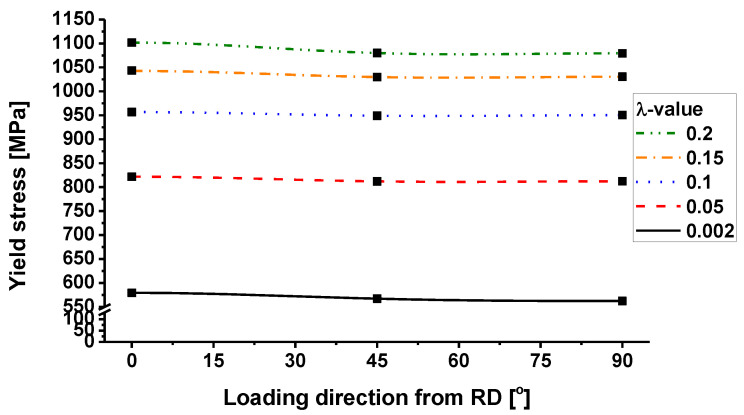
Comparison of uniaxial tensile yield stress evolution between experiments and prediction.

The comparison between the yield stress predicted by the Chen-CQN model for the uniaxial tensile of TRIP780 along RD, TD, and DD and the yield stress obtained from the experiment is given in Figure 10. With the plastic strain increasing, the yield stress values predicted by the model are always in good agreement with the actual uniaxial tensile experimental data, indicating the high accuracy of the Chen-CQN model in characterizing the uniaxial tensile yield stresses at different strain levels.

**Figure 11:**
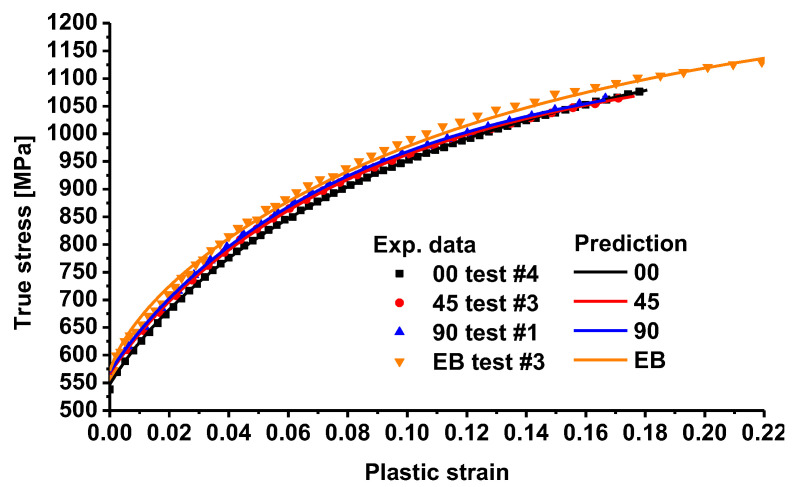
Comparison of stress–strain curves of uniaxial and equibiaxial tension between experiments and prediction.

**Figure 12:**
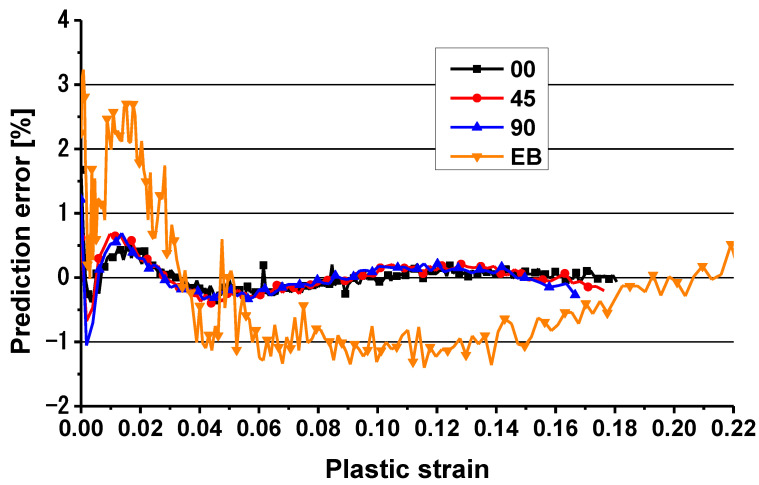
Prediction error of stress–strain curves of uniaxial and equibiaxial tension.

Figure 11 compares the hardening curves of TRIP780 for uniaxial tension along three loading directions (0°, 45°, and 90°) and equibiaxial tension with the prediction of the Chen-CQN yield function. Based on the results shown, it can be seen that the predicted uniaxial tensile anisotropic hardening curves achieve a good fit with the experimental data, while there is a small error between the predicted and experimental data for the equibiaxial tension conditions. The predicted anisotropic hardening curves and the corresponding experimental curves were used to find the errors of the predicted curves, as shown in Figure 12. According to the results shown in the figure, the maximum error of the hardening curves of the Chen-CQN function during the plastic deformation in uniaxial tension were always limited to within 1%. With the increase in strain, prediction error gradually approaches 0%. The maximum error of the prediction curve is at the moment when the plastic deformation of the specimen starts under equibiaxial tension conditions. This is because the hardening curve of the steel fluctuates more in the initial stage of plastic deformation, so the prediction accuracy is reduced. However, as a whole, the error between the predicted curve and the experimental curve of equibiaxial tension was basically kept within 3% from the beginning of the plastic deformation of the steel to the necking. Based on the results of the above diagrams, it is shown that the Chen-CQN function is able to accurately characterize the anisotropic hardening behavior of the TRIP780 steel sheet at different stresses.

## 6. Discussions

In this study, uniaxial tension of dogbone specimens and bulging tests were conducted to characterize the strain hardening behavior of uniaxial tension along different loading conditions and equibiaxial tension. The experimental results show that the strain hardening behavior at different directions and stress states is apparently different by up to about 8%, as shown in Figure 5. The apparent difference cannot be modeled by anisotropic yield functions with isotropic hardening, such as the SY2009 and Yld2000-2d functions.

The anisotropic hardening was modeled by the Chen-CQN model. The convexity of the Chen-CQN yield surface was investigated at different plastic strains. It was observed that the yield surface was convex at different plastic strains. Therefore, the Chen-CQN model can be used to model the anisotropic hardening behavior of TRIP780 steel sheets.

The performance of the Chen-CON model was compared with the SY2009 and Yld20002d functions as well as the experimental results to evaluate the proposed Chen-CON model. It is obvious that the SY2009 function overestimates the yield stress of plane strain by about 3–5% compared to the Yld2000-2d and Chen-CON models. The Yld2000-2d function predicts similar results with the Chen-CON model, but the Yld2000-2d function has different sets of anisotropic parameters at different strains. The prediction accuracy of the Chen-CON model is the highest among the three models. The largest error is less than 3%, as shown in Figure 12. Therefore, the Chen-CON model is recommended to characterize the anisotropic hardening behavior of TRIP780 steel sheets.

## 7. Conclusions

In this paper, hardening experiments of TRIP780 under uniaxial and equibiaxial tension stress states were conducted. The Chen-CQN analytical model was applied to characterize the anisotropic hardening behavior of TRIP780, and the convexity of the yield surface characterized by the function was verified by the dichotomous method. By comparing the prediction of the Chen-CQN function with the experimental data, the results evaluated the prediction accuracy of the Chen-CQN function. The conclusions are as follows:

(1) Both the strength and r-values of TRIP780 steel are directional dependent.
(2) TRIP780 steel has the characteristics of strength anisotropy and plastic deformation anisotropy.
(3) The prediction error of the Chen-CQN function stays within 3%, and the function is capable of accurately describing the plastic deformation of TRIP780 steel under different stress states.
(4) The Chen-CQN function is accurate and flexible when characterizing the anisotropic behavior of TRIP780 steel.

## Figures and Tables

**Table 1:** Chemical compositions of TRIP780 steel %.

C	Si	Mn	Al	N	B	V	Ti
0.19	1.38	≤1.68	0.053	0.028	0.0015	≤0.01	≤0.01

**Table 2:** Coefficients of Swift–Voce function.

Stress State	*r*-Value	*K* [MPa]	*e*_0_	*n*	*A* [MPa]	*B* [MPa]	*C*	α
RD	0.788	1654.04	0.0116	0.2541	1150.66	560.29	11.077	0.4870
DD	0.801	1607.66	0.0105	0.2361	1125.63	572.95	12.357	0.2718
TD	0.963	1615.75	0.0105	0.2356	1119.86	575.81	12.902	0.2635
EB	NA	1436.50	0.0027	0.1726	1246.70	605.87	8.8320	0.3917

**Table 3:** Coefficients of the Yld2000-2d function.

λ	*α*_1_	*α*_2_	*α*_3_	*α*_4_	*α*_5_	*α*_6_	*α*_7_	*α*_8_	m
0.002	0.8463	1.1141	0.8393	0.9869	0.9907	0.8170	0.9713	1.1869	6
0.10	0.8957	1.0643	0.9469	0.9906	1.0000	0.8839	0.9733	1.1060	6
0.20	0.8825	1.0951	0.9521	1.0010	1.0067	0.9132	0.9849	1.1194	6

## Data Availability

Not applicable.
